# The different affinity of the two metal-binding sites of human ferroportin drives outward directionality of transport

**DOI:** 10.1007/s10534-025-00725-2

**Published:** 2025-07-21

**Authors:** Matteo Amadei, Alfredo De Lauro, Fabio Polticelli, Giovanni Musci, Maria Carmela Bonaccorsi di Patti

**Affiliations:** 1 Department of Biochemical Sciences ‘A. Rossi Fanelli’, Sapienza University of Rome, Rome, Italy; 2 Department of Sciences, University Roma Tre, Rome, Italy; 3 Department of Biosciences and Territory, University of Molise, Campobasso, Italy

**Keywords:** Ferroportin, Iron, Cobalt, Major facilitator superfamily, Fluorescence spectroscopy, Metal transport, Membrane transporter

## Abstract

Ferroportin, the only known cellular iron exporter, belongs to the major facilitator superfamily of transporters, which cycle between inward-open, occluded and outward-open conformations to translocate substrates across membranes. Recently reported cryoEM structures of ferroportin identified two metal-binding sites in the central cavity of the protein, with site S1 that includes residues D39 and H43, while site S2 is formed by C326 and H507. Here we have employed fluorescence spectroscopy to evaluate the binding affinity for cobalt of human ferroportin. The results suggest that S2 has a higher affinity for cobalt than S1. Results are discussed in view of available structural data on the outward-open conformation of Fpn and of a novel structural model of the inward-open conformation, obtained with a custom implementation of AlphaFold 2. We propose a mechanism by which the outward flux of iron could be driven by the different affinity of the two sites.

**Supplementary Information:**

The online version contains supplementary material available at 10.1007/s10534-025-00725-2.

## Introduction

Ferroportin (SLC40A1, Fpn) is the only known cellular iron export membrane protein identified so far in mammals and it is essential for physiological regulation of cellular and systemic iron levels (Drakesmith et al. 2015; Jormakka 2023; Galy et al. 2024). Missense mutations of Fpn cause type 4 hereditary hemochromatosis, a dominant form of hemochromatosis with parenchymal and/or reticulo-endothelial iron overload (Pietrangelo 2017; Vlasveld et al. 2019; Uguen et al. 2023).

**Fig. 1:**
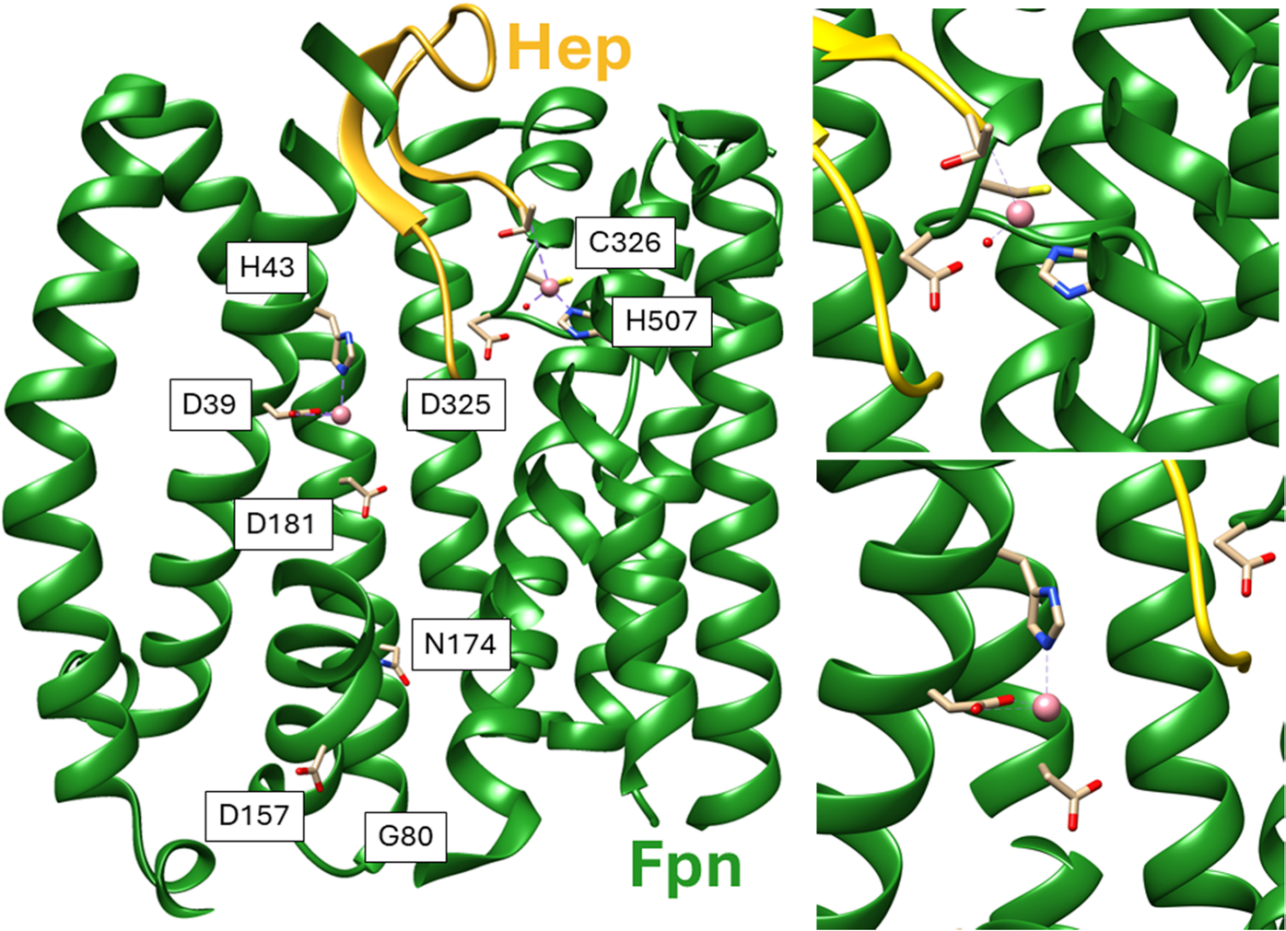
Metal binding sites of Fpn. Cross section of the three-dimensional structure of human ferroportin (Fpn; green ribbon) in complex with hepcidin (Hep; gold ribbon) and cobalt (pink spheres) showing the internal vestibule of the protein in the outward open conformation (PDB: 6WBV). The residues coordinating the cobalt ions and those mutated and/or discussed in this study are shown in stick representation. A close-up view of the iron-binding sites is shown in the two panels on the right

The recently reported cryoEM structures of human Fpn in complex with cobalt and the peptide hormone hepcidin have finally provided a framework for a clearer understanding of metal binding and they have allowed to propose a model of how the peptide regulates the transporter (Billesbølle et al. 2020). In the last years, structures of the protein in the presence of synthetic inhibitors and cations have also been reported (Shen et al. 2023; Wilbon et al. 2023; Lehmann et al. 2023). Fpn belongs to the major facilitator superfamily (MFS) of transporters, which cycle between inward-open, occluded and outward-open conformations to translocate their substrates across membranes (Quistgaard et al. 2016). The protein displays the typical 12-TM MFS fold arranged in two domains, each formed by 6 TM helices (Fig. 1 and S1a). All the structures presently available for mammalian Fpn capture the protein in the outward-open state (Billesbølle et al. 2020; Pan et al. 2020; Shen et al. 2023; Wilbon et al. 2023), except for one in a partially occluded conformation with the inhibitor vamifeport (Lehmann et al. 2023). In the TM2 and TM3 helices there is a sequence motif conserved throughout the MFS superfamily termed the “Motif A”, which is composed by residues G80, D84 and R88 forming an intracellular gate necessary for the stabilization of the outward-open conformation. G80 is necessary to allow tight contact between TM2 and TM11, D84 interacts with the N-terminal end of TM11 while R88, located at the N-terminal end of TM3, forms strong electrostatic interactions with D157, located on TM4 (Fig S1b). At variance with most MFS members, which generally display a single substrate-binding site, two metal-binding sites have been identified in the central cavity of mammalian Fpn (Fig. 1). These sites are about 15 Å apart and have been identified by employing cobalt as a more stable substrate mimic for ferrous iron. Site S1 is closer to the cytosolic face of the protein and it includes residues D39 and H43, while site S2 is formed by C326 and H507 (Billesbølle et al. 2020; Pan et al. 2020; Wilbon et al. 2023). Site-directed mutagenesis and functional studies suggest that S1 is primarily required for iron transport (Bonaccorsi di Patti et al. 2014; Billesbølle et al. 2020). In fact, the S1 mutant D39A is unable to transport iron and it has impaired iron-binding capacity (Bonaccorsi di Patti et al. 2014). On the other hand, S2 may play an additional regulatory role because mutations of the coordinating residues do not impair iron transport but make the protein resistant to hepcidin, the peptide hormone that regulates Fpn function by occluding the transporter and by inducing its internalization and ubiquitination (Aschemeyer et al. 2018). Binding of hepcidin to the central cavity of Fpn in the outward-open conformation involves the S2 site and is strengthened by the concomitant presence at S2 of the divalent metal ion (be it cobalt or iron), which is coordinated also by the carboxy terminus of the peptide (Billesbølle et al. 2020). In contrast with this view, it has been suggested that S2 may have a more prominent role in iron transport than S1 because mutations at S2 had a greater impact on metal transport in a liposome-based assay (Pan et al. 2020; Shen et al. 2023).

An open question regards the molecular details of the mechanism of iron translocation by Fpn; in particular, it is currently unknown what drives the outward directionality of transport. In this work we have employed fluorescence spectroscopy to evaluate the binding affinity for cobalt of wild-type and mutant human Fpn and, also on the basis of the analysis of a novel inward-open structural model, we suggest that the different affinity of the two sites S1 and S2 is responsible for the outward flux of iron.

## Results

### Intrinsic tryptophan fluorescence spectroscopy of wild-type and mutant Fpn

The existence of two substrate-binding sites is a peculiarity of Fpn compared to other MFS membrane transporters. In this work, the possibility that the two iron-binding sites have distinct affinities for the substrate has been explored. Intrinsic tryptophan fluorescence spectroscopy has been used to assess the metal affinity of human Fpn wild-type and a set of mutants. Since attempts to directly measure K_D_ for iron proved to be unfeasible due to the large inner filter effect of ascorbate, that was required to keep iron in the reduced state and avoid precipitation of ferric iron during titration, we employed cobalt as a widely used substitute for ferrous iron due to its stability in the presence of oxygen. As a matter of fact, all the structures of mammalian Fpn with bound metals have been obtained with cobalt and most in vitro transport assays have been carried out with this metal (Billesbølle et al. 2020; Pan et al. 2020; Shen et al. 2023; Wilbon et al. 2023; Lehmann et al. 2023).

**Fig. 2:**
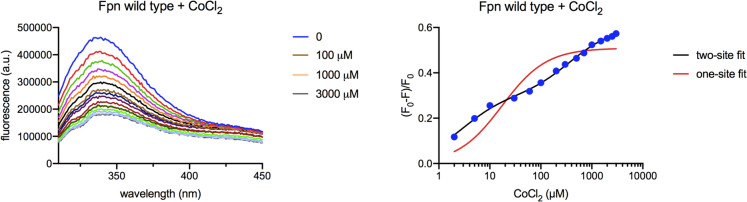
Cobalt titration of Fpn. Wild-type Fpn (500 nM) in MOPS 25 mM, NaCl 150 mM, DDM 0.01% pH 7.0 was titrated with cobalt chloride (2–3000 μM) and emission spectra were recorded with excitation at 295 nm (left panel). Plot (F_0_-F)/F_0_ of mean fluorescence at 333–337 nm vs [CoCl_2_] fit to a one-site or two-site binding equation (right panel)

**Fig. 3:**
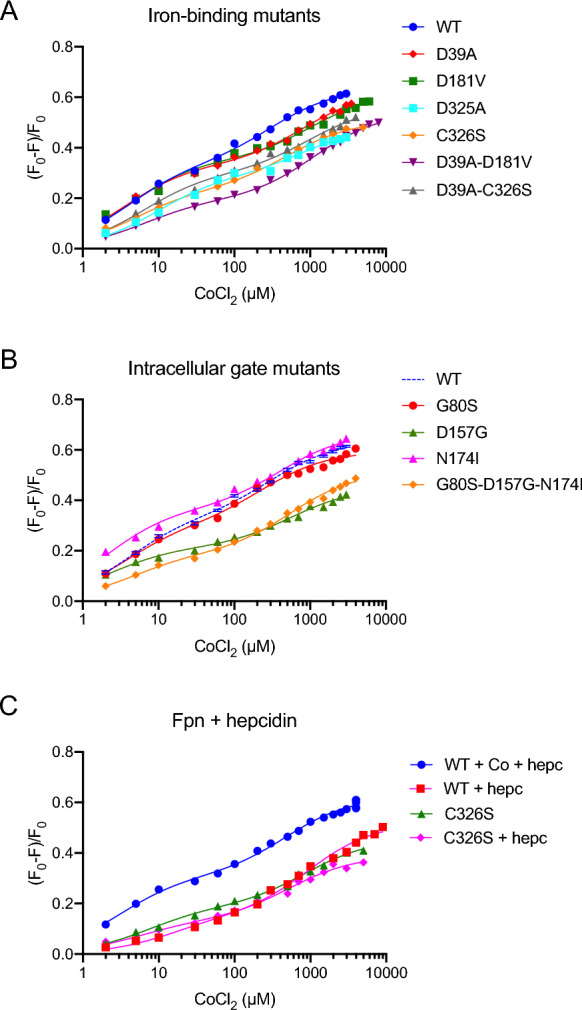
Binding of cobalt to Fpn and effect of hepcidin. Plot (F_0_-F)/F_0_ of mean fluorescence at 333–337 nm vs [CoCl_2_] of wild-type and mutant Fpn (panels A, B). Panel C: plot (F_0_-F)/F_0_ of mean fluorescence at 333–337 nm vs [CoCl_2_] of Fpn (500 nM) with hepcidin (900 nM) added either before or at the end of metal titration. The data were fit to a two-site binding equation

Representative fluorescence spectra of wild-type Fpn titrated with cobalt 2–3000 μM are shown in Fig. 2 (left panel). The (F_0_-F)/F_0_ *vs* [CoCl_2_] curve was fit to a two-site binding model (Fig. 2, right panel); the fit to a one-site model was poor (R^2^ 0.9299, sum of squares 0.0377) compared to that of the two-site model (R^2^ 0.9979, sum of squares 0.0011), suggesting the latter was indeed better. According to this analysis, wild-type Fpn exhibits a high-affinity metal-binding site with K_D high_ close to 5 μM and a low-affinity site with K_D low_ of about 300 μM (Table 1), each contributing about 50% to the fluorescence signal Fig 3.

**Table 1:** K_D_ values for cobalt of human Fpn wild-type and mutants

Fpn	K_D high_ (μM)	(SD)	K_D low_ (μM)	(SD)	n
Wild-type	4.2	1.7	320	89	6
D39A	5.7	2.8	920	200	7
G80S	2.74	0.70	310	120	3
D157G	3.0	2.0	578	69	4
N174I	2.44	0.66	350	160	3
D181V	3.56	0.63	900	240	4
D325A	5.8	2.7	710	250	6
C326S	3.5	1.6	540	120	4
D39A-D181V	7.4	2.8	1030	250	6
D39A-D325A	n.d	–	n.d	–	2
D39A-C326S	6.0	3.6	1070	770	5
G80S-D157G-N174I	5.3	0.78	566	73	3
Wild-type + hepcidin	11.2	2.6	1090	210	3
C326S + hepcidin	5.3	0.7	607	92	3

**Fig. 4:**
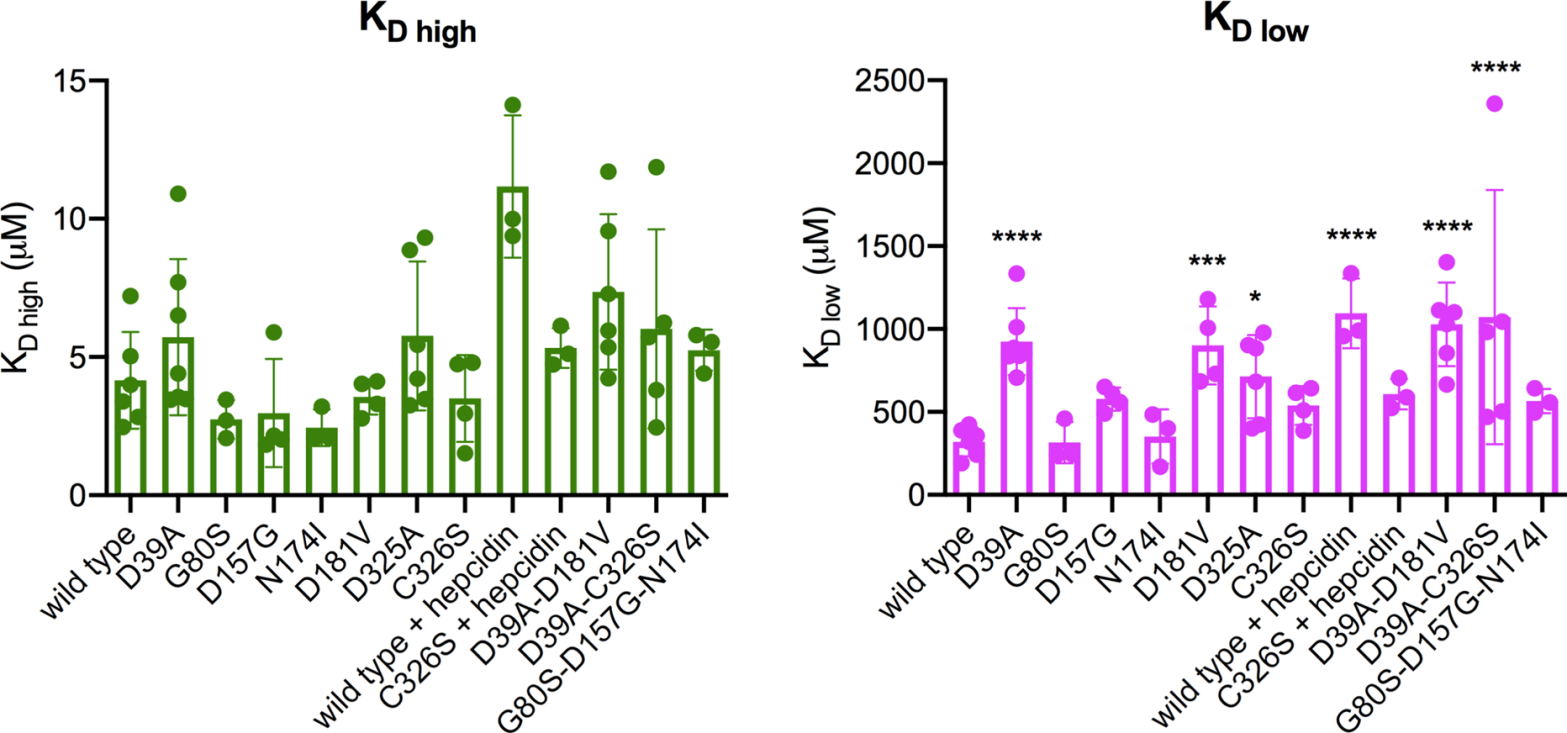
K_D_ values of Fpn for cobalt. Scatter plot with individual K_D_ values. Error bars indicate the SD of the mean. P values were calculated by two-way ANOVA with Dunnett’s post hoc test; **** p < 0.0001; *** p < 0.0005; * p < 0.05 *vs* wild-type

The mean of the K_D_ values shown in Fig. 4, obtained by individual curve fits, is reported. *SD* standard deviation, *n* number of independent replicates; *n.d.* not determined

Next, two sets of mutants either targeting the metal-binding sites or focusing on residues forming the intracellular gate that stabilizes the outward open conformation of Fpn have been analyzed (Fig. 1). Metal-binding site mutants include the S1 mutant D39A, and S2 variants C326S and D325A. While D39A is unable to transport iron and has impaired iron-binding capacity (Bonaccorsi di Patti et al. 2014), substitution C326S at site S2 causes resistance to hepcidin without impairing the iron export ability of Fpn (Fernandes et al. 2009; Aschemeyer et al. 2018). D325 has been shown to be involved in iron binding at S2 through a water-mediated contact in the presence of hepcidin (Billesbølle et al. 2020). Notably, the D325A mutant displays significantly decreased iron export capacity (Bonaccorsi di Patti et al. 2014; Deshpande et al. 2018; Le Tertre et al. 2021).

Among mutations identified in hemochromatosis patients, D181V was selected because it causes inability to transport iron and the protein has reduced iron-binding ability (Bonaccorsi di Patti et al. 2014; Praschberger et al. 2014). On the other hand, pathogenic loss of function mutants G80S, D157G and N174I are part of a network of interactions thought to stabilize the outward-open conformation of Fpn acting as an intracellular gate (Tortosa et al. 2017; Guellec et al. 2019). Since the outward-open conformation has been proposed to represent the resting state of Fpn in solution (Billesbølle et al. 2020; Amadei et al. 2023; Jormakka 2023), disruption of the intracellular gate could cause the protein to adopt a prevalent inward-open or occluded conformation, allowing access to these forms of Fpn.

The fluorescence spectra of the described mutants are shown in Figs S2 and S3; representative (F_0_-F)/F_0_ *vs* [CoCl_2_] curves are displayed in Fig. 3 with the corresponding fit to the two-site model. All K_D_ values are presented in Fig. 4 and Table 1. For all the Fpn mutants the K_D high_ values show no statistically significant change compared to wild-type Fpn. The K_D low_ values evidence that the low-affinity site is compromised in Fpn D39A, D181V and the double mutant D39A-D181V, with a statistically significant, about threefold increase of K_D low_. The K_D low_ of Fpn D325A is increased over twofold. The S1-S2 mutant D39A-C326S shows very high variability, mostly affecting K_D low_. The D39A-D325A mutant appears to be unable to bind cobalt, supporting the specificity of the fluorescence response, as also assessed by titration of free tryptophan (Fig S5). On the other hand, K_D_ values of Fpn G80S, N174I and C326S are not significantly affected. The triple mutant G80S-D157G-N174I displays an increase in K_D high_, although not significant, compared to the single mutants, and K_D low_ close to D157G.

The effect of hepcidin has been evaluated by cobalt titration of Fpn in the presence of the peptide (Fig. 3C). The results show that hepcidin causes a marked increase of both K_D high_ and K_D low_, reducing the ability of wild-type Fpn to bind the metal (Table 1). When the peptide is added to the protein saturated with cobalt, the fluorescence signal is essentially unchanged, suggesting that hepcidin does not displace cobalt (Fig. 3C). Preincubation with hepcidin has no significant impact on both K_D_ values for cobalt of the Fpn C326S mutant (Fig. 4 and Table 1).

### Structural model of the inward-open conformation of Fpn

**Fig. 5:**
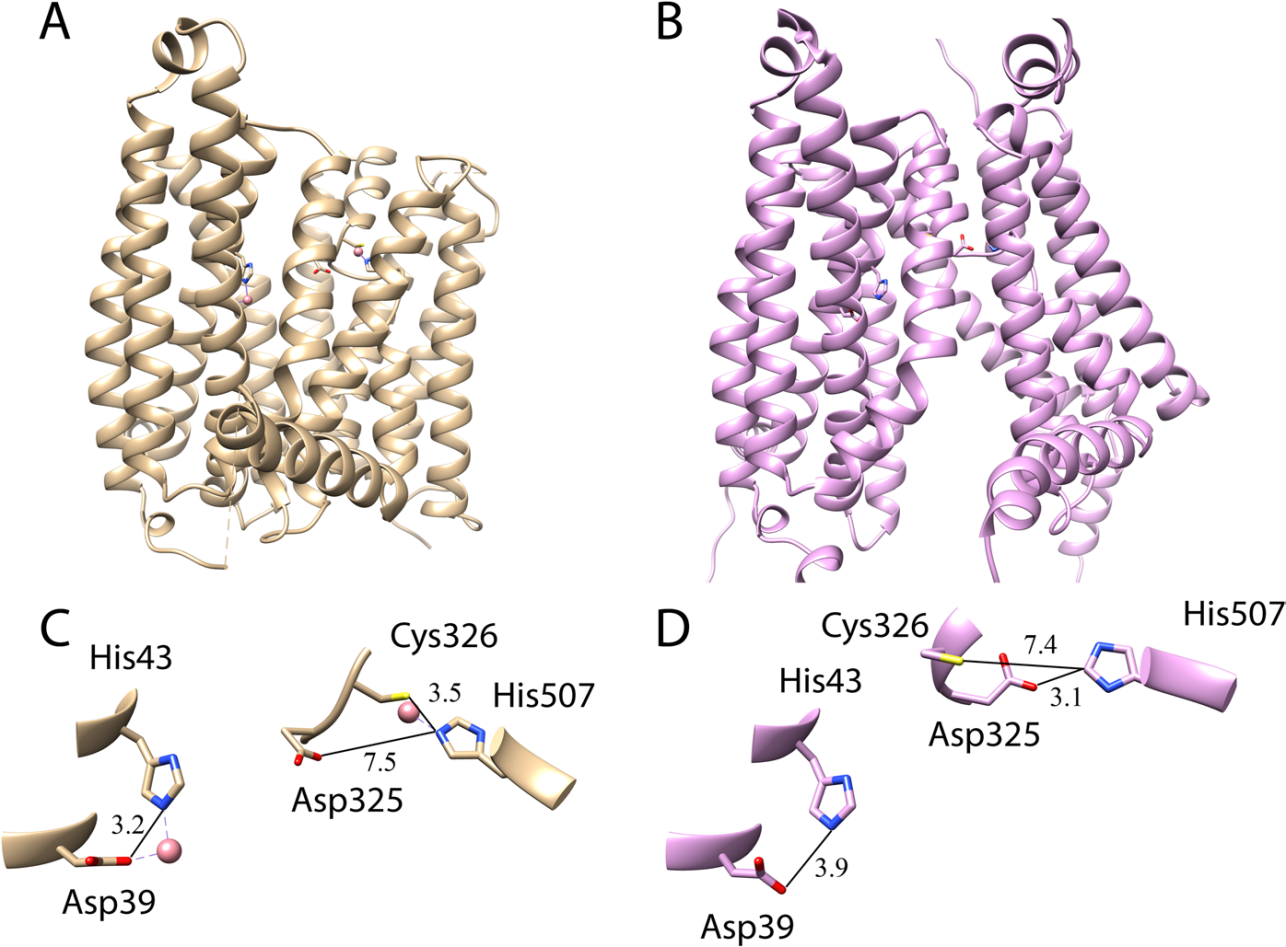
Outward-open and inward-open conformations of Fpn. **A**) Schematic representation of the three-dimensional structure of Fpn in the outward-open conformation (PDB code 8DL8) and **B**) of the structural model of the inward-open conformation obtained with ColabFold (this work). **C**) and **D**) detailed view of the respective metal binding sites

The structural model of the inward-open conformation of Fpn has been obtained by a local implementation of ColabFold that allows to change various AlphaFold input parameters, including the max_msa value. This allows AlphaFold to extract from the MSA different, conformation-dependent, residue-residue co-evolution signals and to model different structural conformations of the same protein (see Methods Section for details). The value max_msa = 512:1024 yielded the inward-open conformation of Fpn shown in Fig. 5. As can be seen from the comparison of the outward-open cryo-EM structure of Fpn bound to Co^2+^ (PDB code 8DL8; Fig. 5A), the structural model obtained is fully inward-open (Fig. 5B). Superposition of the model with the inward-open structure of *Bdellovibrio bacteriovorus* Fpn (PDB code 5AYO) shows a Cα root mean square deviation value of 2.04 Å calculated on the TM helices (Fig S6).

Comparative analysis of the two conformations confirms that Fpn operates through a rigid body movement of the N- and C-terminal domains, as expected for the rocker-switch movement hypothesized for most members of the MFS (Quistgaard et al. 2016). In fact, individual fit of the N-terminal and C-terminal domains of the two conformations, excluding loops, yields root mean square deviation values around 0.7 Å.

In the outward-open cryo-EM structure of Fpn bound to Co^2+^, a metal ion is bound by residues D39 and H43 (site S1), located in the N-terminal domain of the protein, while a second metal ion is bound by C326 and H507 (site S2) and is closer to the extracellular face of the protein (Billesbølle et al. 2020; Pan et al. 2020; Wilbon et al. 2023) (Fig. 5C). Interestingly, in the inward-open structural model, the position and relative orientation of D39 and H43 are almost identical to those of the outward-open structure while the distance C326-H507 increases from 3.5 Å (in the outward-open conformation) to 7.4 Å and, on the contrary, the D325-H507 distance decreases from 7.5 Å (in the outward-open conformation) to 3.1 Å. This result suggests that while S1 is not affected by the inward-outward conformational transition, S2 in the outward-open conformation of the protein may form only after the conformational change takes place.

## Discussion

Prompted by a comment of an anonymous reviewer of a previous paper, we have reevaluated the affinity of Fpn for cobalt, extending the earlier analysis (Amadei et al. 2023). Quenching of tryptophan fluorescence is linked to metal binding, as suggested by lack of response upon binding of hepcidin to Fpn (Fig S7) or for the double mutant D39A-D325A (Fig S5). The specificity of the fluorescence response was evaluated also by titration of free tryptophan with cobalt (Fig S5). The quenching observed at high cobalt concentration shows a different trend compared to Fpn and it may be attributed to formation of a complex of free Trp with metals (Tabak et al. 1989). No evidence for direct binding of cobalt to Trp residues in Fpn is available. Thus, we hypothesize that the tryptophan fluorescence response is due to a conformational transition of Fpn caused by metal binding, possibly pushing the protein towards an occluded state, as proposed in (Amadei et al. 2023). This would be coupled to local rearrangements that lead to quenching of Trp fluorescence, but until an experimentally determined inward-open structure of Fpn with bound metal(s) becomes available it is hard to disclose the contribution of each Trp residue to the cobalt-dependent spectral changes. Some considerations can nevertheless be made. Human Fpn contains 10 tryptophan residues distributed throughout the structure. Residue W42 is close to site S1 and it has been shown to be critical for iron export (Le Gac et al. 2013), W158 is just after D157 at the intracellular gate and substitutions W158C and W158L cause loss of function associated to pathology (Vlasveld et al. 2019; Uguen et al. 2023). The other 8 tryptophan residues are distant from the metal binding sites and are within TM helices, except for W299 in the intracellular loop between TM6 and TM7. Superposition of our inward-open model onto the outward-open structure (PDB code 8DL8) indicates that W82, W158 and W224 are located on TM helices that experience substantial conformational changes, compared to more limited position and/or rotamer variations for most other Trp (including W42). Hypothetically, cobalt binding to S1 could be associated to quenching of W42 while binding to S2 may produce a larger conformational change, involving other tryptophan residues (W158 at the intracellular gate, together with W82 and W224?). In support of this hypothesis, rearrangements at S2 appear to be more extensive than at S1 when comparing the outward-open structure to the inward-open model. Cobalt binding to other sites cannot be ruled out, but it appears unlikely because they would probably have been identified in the cryoEM structures that were obtained by soaking Fpn in 0.6–10 mM CoCl_2_ (Billesbølle et al. 2020; Pan et al. 2020; Wilbon et al. 2023). We cannot formally exclude that other metal sites may be found in the large loops missing from the experimentally determined Fpn structures, but these loops are predicted to be quite flexible making metal binding improbable.

Given the limitations of the fluorescence assay, that does not tie the spectral changes directly to S1 or S2, the cobalt titrations nevertheless suggest the existence of metal-binding sites with differing affinities. The finding that two different K_D_ values are observed for the metal-binding sites of Fpn is novel and it allows to reconcile some contrasting data reported in the literature. Actually, if assays are carried out with a metal concentration of 100 μM or lower, essentially only the high-affinity site will contribute. In fact, the high-affinity K_D high_ is fully in line with our previously published value, that was obtained by titration with cobalt 2–50 μM (Amadei et al. 2023), and with the affinity estimation obtained in a *Xenopus* oocyte expression system (Mitchell et al. 2014). Intriguingly, ITC analyses reported by different groups observed a single K_D_ for cobalt of the same order of magnitude of our low-affinity K_D low_ (Deshpande et al. 2018; Pan et al. 2020; Shen et al. 2023). In these studies, however, the presence of additional metal ions (i.e. Ca^2+^) as well as other factors such as the detergent used to solubilize Fpn may explain the different values for number and strength of the cobalt-binding site(s), also considering the likely consequent heterogeneity (i.e. inward/outward) in protein conformations (Pan et al. 2020).

Regarding the assignment of the two K_D_ values to either S1 or S2, the results obtained with the Fpn mutants provide some potential indications. The threefold increase in K_D low_ obtained with Fpn D39A (S1 mutant) and D181V suggests that S1 may be tentatively assigned as the low-affinity site. The report that the D39A mutant was found to be similar to wild-type Fpn in a liposome-based transport assay performed with Fe^2+^ 10 μM (Li et al. 2020) supports our suggestion that S1 is the low-affinity site because at this low concentration of metal only the behavior of the high-affinity site can be observed. The naturally occurring mutation D181V causes ‘ferroportin disease’ due to failure of Fpn to efficiently export iron, and decreased iron-binding capacity (Bonaccorsi di Patti et al. 2014; Praschberger et al. 2014). Residue D181 is located on TM5 closer to the cytosolic side and about 8.5 Å away from D39 in the outward-open structure of Fpn (PDB code 6W4S), but its side chain moves nearer to D39 in the occluded form (7.1 Å; PDB code 8C03) and even more in the structural model of the inward-open form here described (6.8 Å). This suggests that D181 may take part in the intracellular entrance path of iron to site S1. The double mutant D39A-D181V is similar to D39A, suggesting that this aspartate residue plays a more prominent role in iron binding.

Somewhat unexpectedly, the S2 site mutant C326S appears to maintain full metal binding capacity. However, this result is consistent with the observation that the C326S mutant exports iron as efficiently as the wild-type protein (Fernandes et al. 2009; Aschemeyer et al. 2018). A possible explanation is that coordination of the metal at the S2 site is more complex, with other residues (e.g., D325 and T320) participating and compensating for the absence of C326, as suggested by the structure of the complex of Fpn with cobalt and hepcidin (Billesbølle et al. 2020). The definite importance of C326 for hepcidin binding is confirmed by the lack of effect on K_D_ by preincubation of the C326S mutant with the peptide, at variance with the result obtained with wild-type Fpn. Support for the hypothesis that S2 may be the high-affinity site comes from MD simulations that indicated that ferrous iron, initially positioned randomly in bulk solvent, localized close to D325, D504 and H507 at the S2 site, only occasionally contacting D39 of S1 (Billesbølle et al. 2020).

Fpn D325A exhibits K_D_ values similar to the S1 site mutant D39A, with twofold higher K_D low_ compared to wild-type Fpn. In the presence of hepcidin, residue D325 contributes to binding of iron at S2 through a water-mediated contact, while in the absence of metal the side-chain of D325 is much more mobile (Billesbølle et al. 2020). Intriguingly, modeling of the inward-open conformation of human Fpn predicts that D325 could participate in an alternative ion binding mode with H507 at the S2 site, suggesting that this aspartate residue may compensate for mutations at C326. Remarkably, the double mutant D39A-D325A appears to be essentially unable to bind cobalt, with a greater defect in metal binding than would be expected through the combined effects of the single mutants. Whether this is due to direct loss of binding of the metal or to an impact on the capacity of the protein to change conformation remains to be established. A critical role of residue D325 for the stability of Fpn was confirmed recently (Le Tertre et al. 2021).

Since the two aspartate residues are embedded deeply in the central cavity of Fpn, the higher variability in both K_D high_ and K_D low_ values observed for the D39A and D325A mutants (and the two D39A-containing double mutants) could be due to local instabilities that, especially for D325A mutant, increase the local flexibility and induce a conformational heterogeneity of the protein with subtle changes in metal affinity.

Pathogenic loss-of-function substitutions G80S, D157G and N174I are all hypothesized to alter the TM5-TM10 intracellular gate that stabilizes the outward-open conformation of Fpn (Tortosa et al. 2017; Guellec et al. 2019), presumably impairing the inward-open to outward-open transition, and leading to strongly decreased ability to translocate iron. However, the G80S, D157G and N174I mutants retain iron-binding capacity similar to the wild-type protein. The triple mutant G80S-D157G-N174I shows a K_D high_ comparable to wild-type Fpn and a K_D low_ value similar to the D157G mutant, possibly pointing to a more critical role for this latter residue in maintaining the integrity of the intracellular gate.

On the basis of the assumptions outlined above, we propose that iron export by Fpn may be driven by the different affinity of the two metal binding sites: S1 would capture iron when intracellular levels are high and translocate it to site S2 driving outward directionality of transport. In support of this model, the tarsier Fpn S2 mutant C326A-H508A had a significantly slower rate of metal transport in a liposome-based assay, while the S1 mutant D39A-H43A showed only a modest decrease (Pan et al. 2020). Once bound to S2 the metal would be held strongly enough to require a ferroxidase for release, avoiding escape of potentially toxic ferrous iron.

The ferroxidases ceruloplasmin and hephaestin, enzymes that partner with Fpn to safely generate the ferric iron that is bound by transferrin for distribution to all tissues (Helman et al. 2023; Amadei et al. 2025), exhibit two K_m_ values for ferrous iron, which differ by approximately two orders of magnitude (ceruloplasmin: K_m1_ 0.6 μM and K_m2_ 50 μM (Osaki 1966); hephaestin: K_m1_ 3.5 μM and K_m2_ 107 μM (Vashchenko and MacGillivray 2012)), with the low K_m1_ consistent with the proposed transfer of the metal from S2 of Fpn. Therefore, the ferroxidase would create an iron gradient from Fpn to the final recipient, transferrin, coupling oxidation and dissociation and providing a further driver of outward flow of iron. In line with this prediction, inclusion of ceruloplasmin and apo-transferrin in the efflux medium of *Xenopus* oocytes expressing Fpn increased the efflux rate constant for iron (Mitchell et al. 2014).

The higher affinity of S2 would also hold iron in place facilitating hepcidin binding to Fpn. This would provide a molecular explanation as to why if a ferroxidase activity is lacking, such as in aceruloplasminemia, iron is trapped on Fpn and the transporter is bound by hepcidin, internalized and degraded (De Domenico et al. 2007). We show here that hepcidin binding to cobalt-free Fpn remarkably increases K_D_ values for both metal-binding sites. Thus, when hepcidin levels are high, the peptide can bind also in the absence of iron and it reduces the capacity of Fpn to capture the metal. It would be interesting to test if the synthetic inhibitor vamifeport, which binds to the central cavity of Fpn in a site overlapping that of hepcidin (Lehmann et al. 2023), produces analogous effects on K_D_ values.

When iron moves to S2 either a second iron ion can enter S1, or the site is empty until the iron in S2 is released. The presence of cobalt at both sites in all the published cryoEM structures of Fpn with metals suggests that simultaneous occupancy of S1 and S2 is feasible. A proton gradient has been proposed to be coupled to iron transport; however, conflicting data have been reported indicating that Fpn may be a proton symporter (Li et al. 2020) or an antiporter (Pan et al. 2020). In the latter case, H43 and H507 may be protonated in the absence of iron due to the vicinity of D39 and D325, and become deprotonated when iron is bound.

The low affinity of S1 would pose a question concerning the efficiency of Fpn-mediated export of iron in a physiologic cellular context, where very low levels of free iron are found in the cytosol. It is possible that the interaction with iron-loaded PCBP2, the chaperone that acts as intracellular iron donor to Fpn (Yanatori et al. 2016), increases the affinity of S1. In line with this assumption, it was shown that Fe-PCBP1 and Fe-PCBP2 bound to ferritin with over ten-fold higher affinity than ferrous iron alone (Leidgens et al. 2013). Anyhow, even in the presence of PCBP2, S1 is still expected to display a much lower affinity than S2 thus making the proposed mechanism still realistic.

For the high-affinity site, K_D high_ is in the μM range, consistent with the moderately weak binding required for rapid transport of substrates across the membrane. It is important to point out that it is possible that our (and others’) metal-binding studies reflect the properties of the outward-open conformation of Fpn, as this is likely the resting state of the protein in solution (Tortosa et al. 2020; Jormakka 2023). In the inward-facing form, which is responsible for binding intracellular iron, the status of the metal binding sites may differ. It is tempting to assign the K_D_ values obtained for the conformationally-restricted G80S, D157G and N174I mutants and the triple mutant to the protein in the inward-open conformation. However, until an experimental three-dimensional structure of human Fpn in the inward-open state with bound metal(s) becomes available, this proposal should be viewed with caution and the hypothesis that the inward-open conformation may possess higher affinity for iron and possibly additional amino acid residues might be involved in metal coordination should not be disregarded.

In this respect and as a final remark, the results here reported point to a redundancy and robustness of the iron transport mechanism of Fpn, with the involvement of residues other than D39, H43, C326 and H507, that may transiently coordinate ferrous iron along the transport path (i.e., D181 and D325) and, at least partially, compensate for the lack of one of the primary ligands.

## Methods

### Expression and purification of recombinant human Fpn

Flag-tagged human Fpn wild-type and mutants were expressed in the yeast *Pichia pastoris* and purified by affinity chromatography on anti-flag agarose G1 (GenScript) as described (Amadei et al. 2023). The purified protein was dialyzed against MOPS 25 mM, NaCl 150 mM, DDM 0.01% pH 7.0 prior to titrations. Millipore Ultra (10 K) devices were used to concentrate purified Fpn when required. Protein content was measured with the microBCA assay (ThermoFisher) or by absorbance at 280 nm (ε_280_ 78000 M^−1^ cm^−1^). Purity and aggregation of Fpn were assessed by SDS-PAGE and size-exclusion chromatography (Fig S8).

### Fluorescence spectroscopy

Fluorescence spectra were obtained using a Horiba Jobin Yvon Fluoromax-3 spectrofluorometer. Protein samples were diluted (typically to 500 nM) in MOPS 25 mM, NaCl 150 mM, DDM 0.01% pH 7.0 and titrated with CoCl_2_ 2–9000 µM. After each addition of metal, the samples were allowed to equilibrate for 5 min in the cuvette before recording of the spectra. To test the effect of hepcidin, 500 nM Fpn was incubated for 10–15 min with 900 nM human hepcidin-25 (Bachem) either before addition of CoCl_2_ or at the end of the titration with CoCl_2_. Excitation wavelength was 295 nm and the emission spectra were recorded between 310 and 450 nm at 25 °C (slit width 5 nm for both excitation and emission). Titration data were analyzed with Prism 8 (GraphPad) employing the two-sites specific binding equation:

$$ {\text{Site1 }} = {\text{ BmaxHi}}*{\text{X}}/\left( {{\text{KdHi }} + {\text{ X}}} \right) $$

$$ {\text{Site2 }} = {\text{ BmaxLo}}*{\text{X}}/\left( {{\text{KdLo }} + {\text{ X}}} \right) $$

$$ {\text{Y }} = {\text{ Site1 }} + {\text{ Site2}} $$

with Y being (F_0_-F)/F_0_, X the variable [CoCl_2_] and Bmax the maximal specific signal. To improve reliability, the mean of the fluorescence values from 333 to 337 nm was employed to construct the (F_0_-F)/F_0_ vs [CoCl_2_] curves. Three to seven replicates with protein samples from different purifications were obtained for wild-type Fpn and each mutant. P values were calculated by two-way ANOVA with Dunnett’s post hoc test (alpha: 0.05).

The UV–Vis absorption spectrum of CoCl_2_ 3 mM is shown in Fig S9. Titration of PNP oxidase (generously provided by Dr. Claudio Graziani, Dept. Biochem. Sci., Sapienza University) and free tryptophan with CoCl_2_ are provided in Fig S5 to demonstrate the specificity of the fluorescence response.

### Modeling of the inward-open Fpn structure

The inward-open structural model of human Fpn (Uniprot ID: Q9NP59) has been obtained by a local implementation of ColabFold (Jumper et al. 2021; Mirdita et al. 2022). ColabFold allows to vary different parameters, including the max_msa parameter. This parameter is the combination of the ‘max_msa_clusters’ and ‘max_extra_msa’ parameters. The former specifies the number of sequence clusters provided to the AlphaFold 2 neural network. The latter specifies the number of extra sequences used to compute additional statistics. Thus, max_msa allows to set the number of clusters of the multiple sequence alignments (MSAs) that are used by AlphaFold 2 to perform the structure modeling. In fact, AlphaFold, given the input amino acid sequence, performs a MSA on a dataset of sequences and generates a multiple alignment of ~ 50000 sequences. For the next steps only a subset of these sequences is used to extract the features necessary for structure modeling. Using different values of the max_msa parameter, it is possible to set the number of clusters that will be supplied to AlphaFold. This allows AlphaFold to extract from the MSA different, conformation-dependent, residue-residue co-evolution signals and to model different structural conformations of the same sequence (Del Alamo et al. 2022; Wayment-Steele et al. 2024) providing insight into the transport mechanism of membrane transporters (Pasquadibisceglie et al. 2022). With this approach, various simulations have been carried out varying the max_msa parameter value, obtaining the inward-open conformation of Fpn with a value of max_msa = 512:1024. The pLDDT plot is shown in Fig S10. The model is available in ModelArchive at https://www.modelarchive.org/doi/10.5452/ma-6m2tq

## Supplementary Information

Below is the link to the electronic supplementary material.

Supplementary file1 (PDF 3085 KB)

## Data Availability

All data are contained within the manuscript.
