# Supplementary material for: The different affinity of the two metal-binding sites of human ferroportin drives outward directionality of transport
Source: Biometals. 2025 Jul 21;38(5):1631–42. doi: 10.1007/s10534-025-00725-2 (PMC12594662; doi:10.1007/s10534-025-00725-2)
Supplement: Supplementary file 1 — Supplementary file1 (PDF 3085 KB) [file 10534_2025_725_MOESM1_ESM.pdf]

Supplementary figures

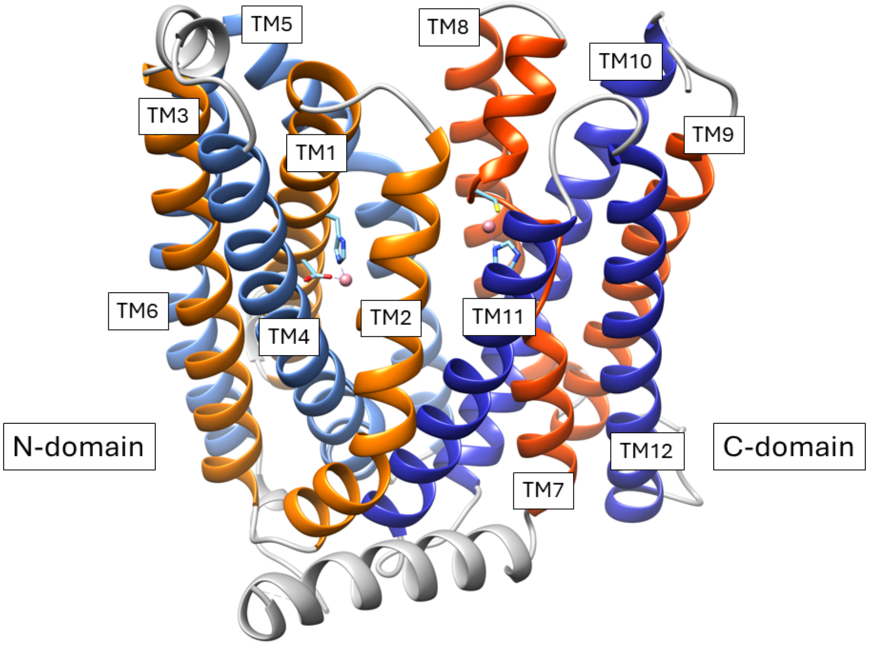

Figure S1a. Overall structure of human Fpn (PDB code 8DL8) (left). Helices are numbered and the first and second repeat of the N-domain are in orange and light blue, respectively. The corresponding repeats of the C-domain are in red and blue. The large intracellular region connecting the two domains is in gray. Metal binding residues are shown in stick representation, and the cobalt ions are shown as pink spheres.

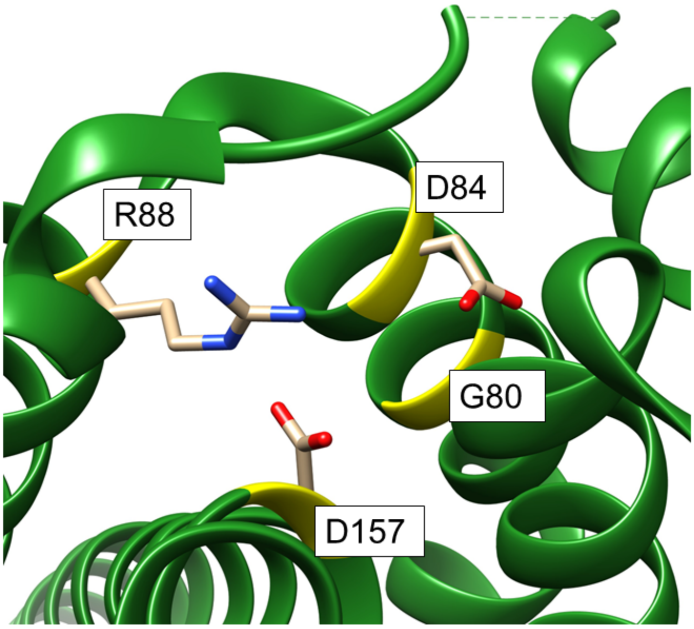

Figure S1b. Schematic representation of the relative arrangement of the residues forming the "Motif A" in Fpn (G80, D84, and R88). D157, forming strong electrostatic interactions with R88, is also shown.

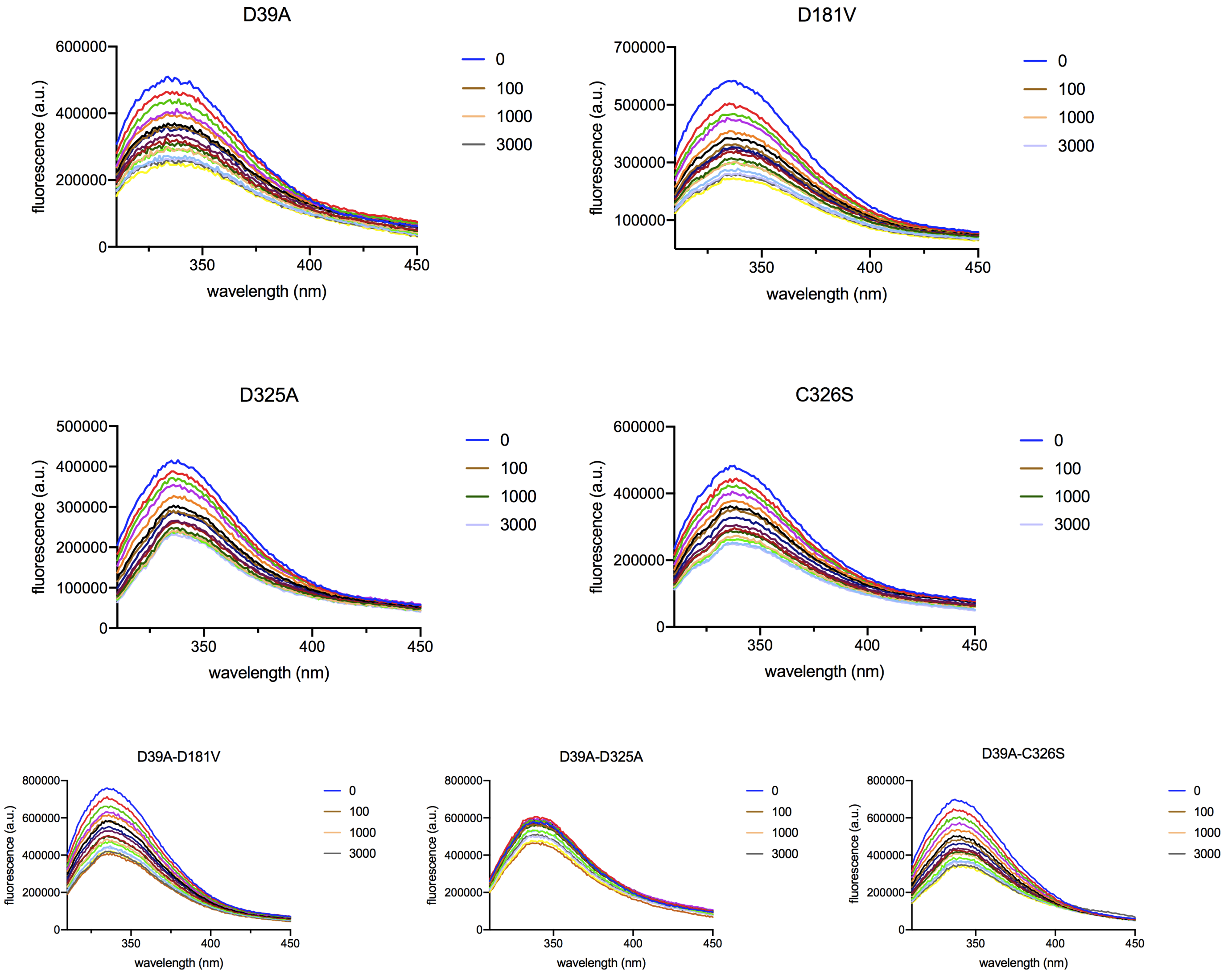

Figure S2. Cobalt titration of Fpn iron-binding site mutants. Fpn (500 nM) in MOPS 25 mM, NaCl 150 mM, DDM 0.01% pH 7.0 was titrated with cobalt chloride (2-3000 µM) and emission spectra were recorded with excitation at 295 nm.

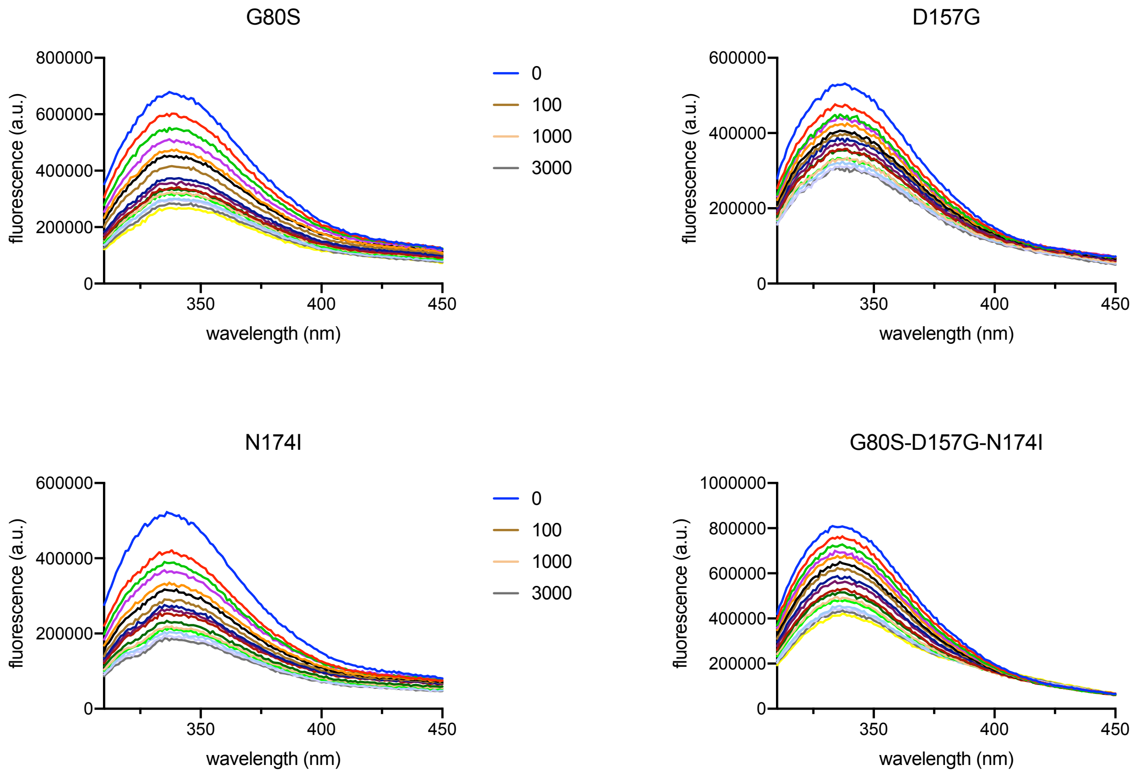

Figure S3. Cobalt titration of Fpn intracellular gate mutants. Fpn (500 nM) in MOPS 25 mM, NaCl 150 mM, DDM 0.01% pH 7.0 was titrated with cobalt chloride (2-3000 µM) and emission spectra were recorded with excitation at 295 nm.

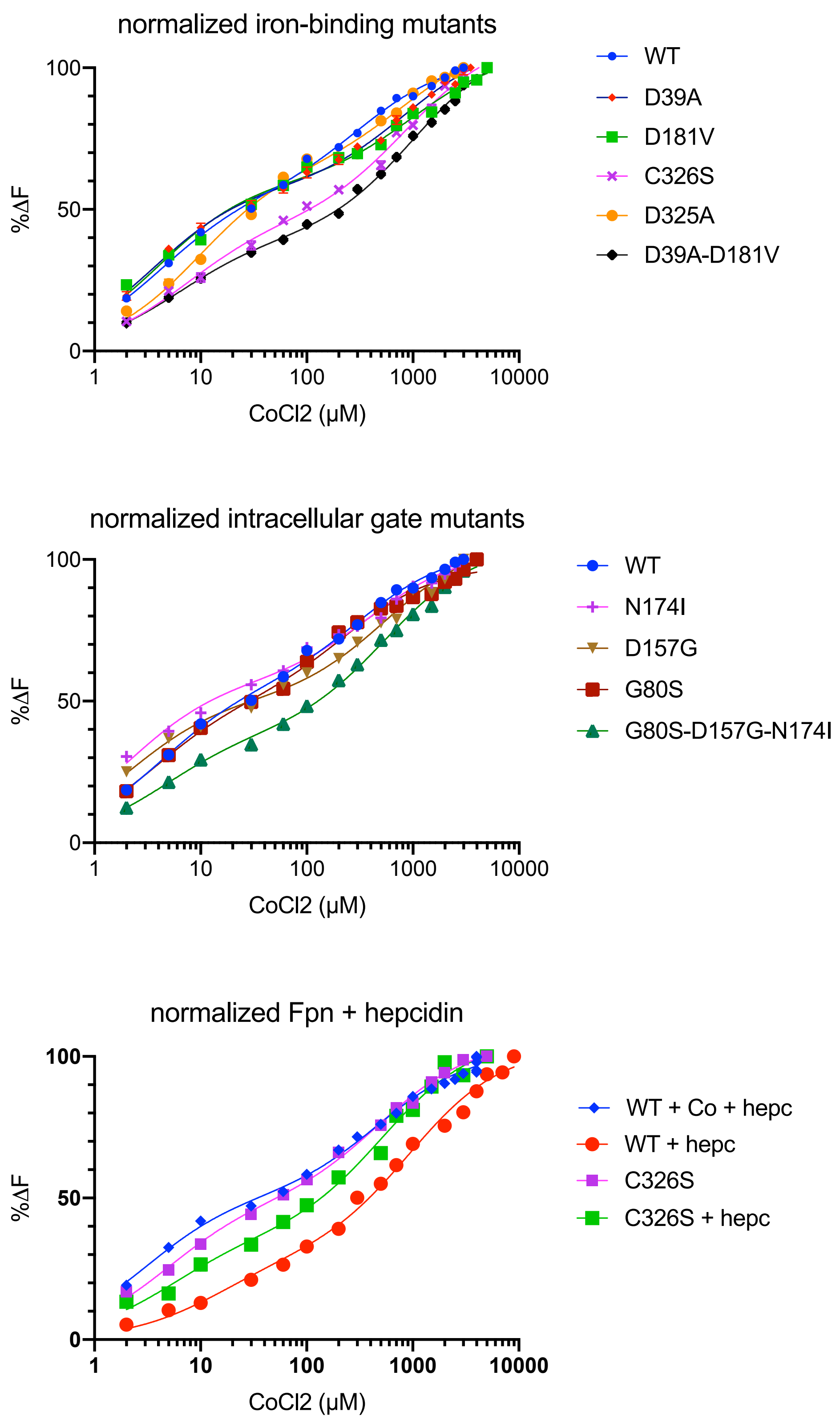

Figure S4. Normalized 0-100% response for Fpn wild-type and mutants shown in Figure 3.

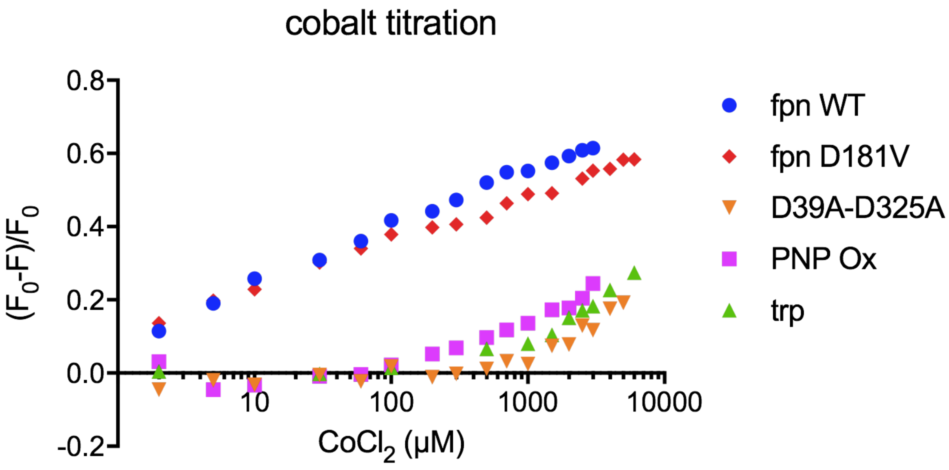

Figure S5. Plot $(F_0-F)/F_0$ of mean fluorescence at 333-337 nm vs $[CoCl_2]$ of Fpn WT, D181V and D39A-D325A compared to PNP oxidase and free tryptophan.

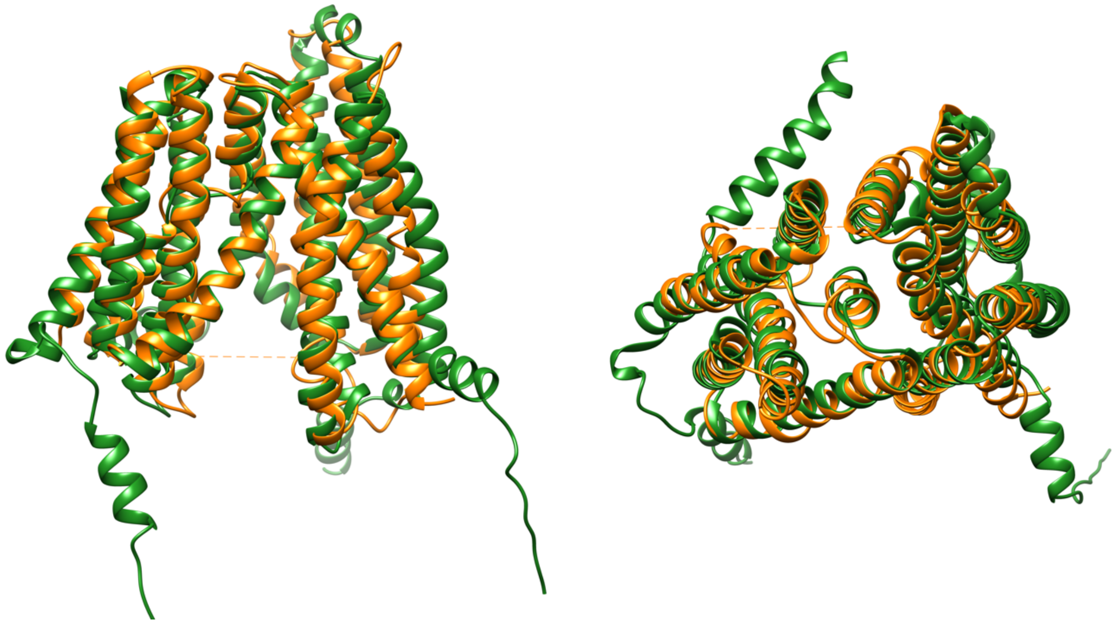

Figure S6. Superposition of the inward open model of human Fpn (green) with the structure of *Bdellovibrio bacteriovorus* Fpn (PDB code 5AYO) (gold).

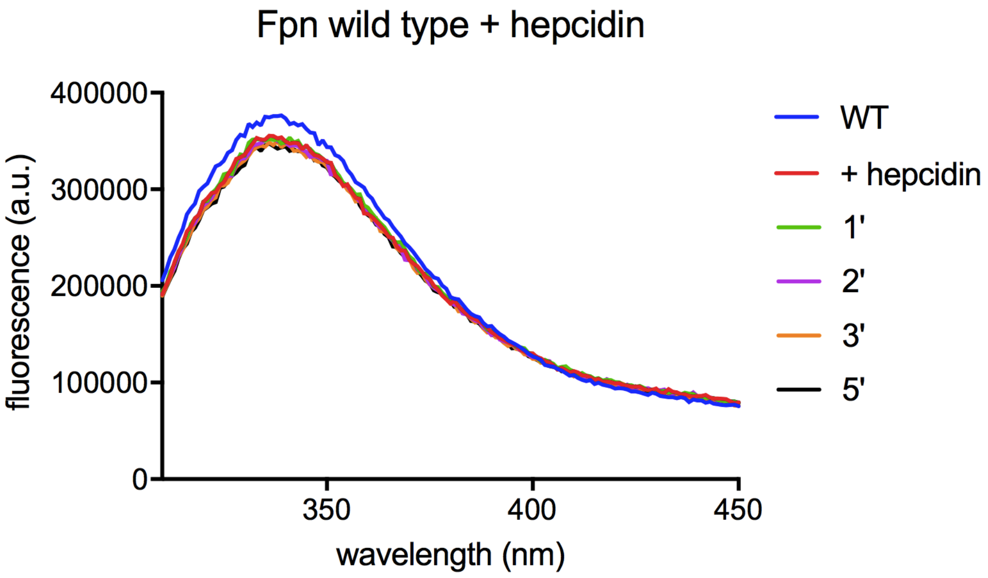

Figure S7. Binding of hepcidin to Fpn. Hepcidin (900 nM) was added to Fpn (500 nM) in MOPS 25 mM, NaCl 150 mM, DDM 0.01% pH 7.0 and emission spectra were recorded with excitation at 295 nm.

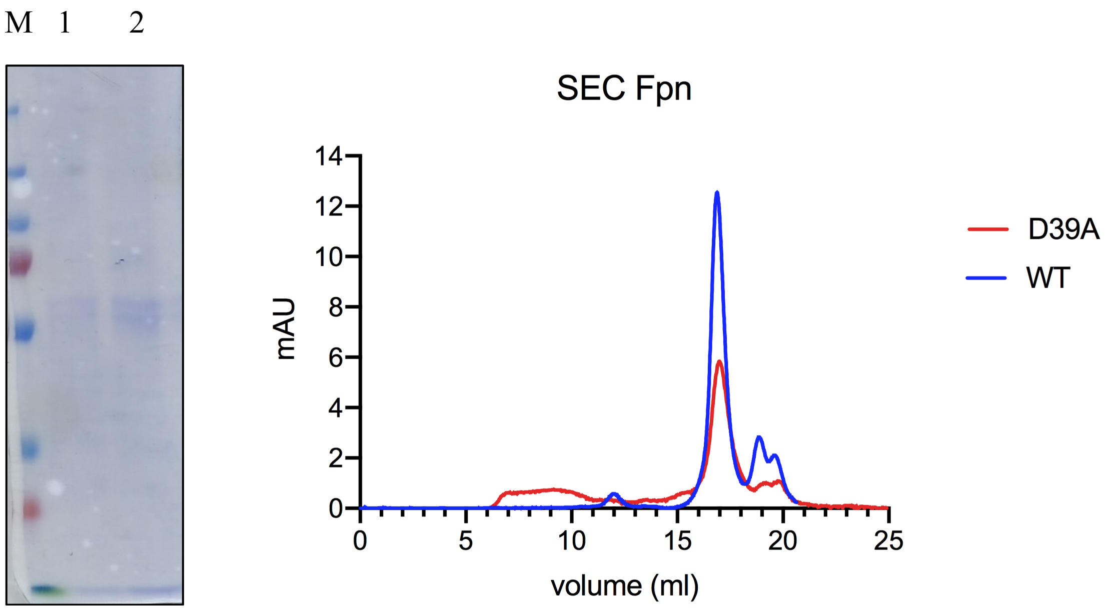

Figure S8. SDS-PAGE and SEC analyses of Fpn. Lane M: M_r markers; lane 1: D39A; lane 2: wild type. SEC was performed on Sephadex G200 (10/300) at 0.5 ml/min in MOPS 25 mM, NaCl 150 mM, DDM 0.01% pH 7.0.

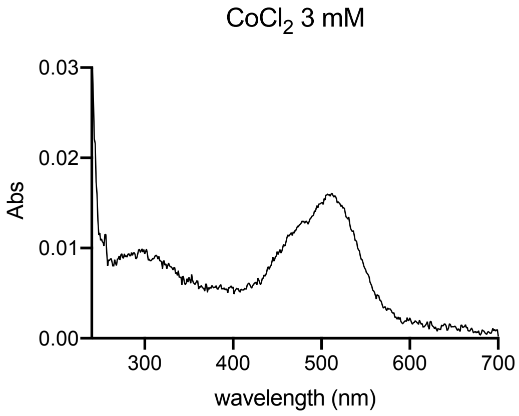

Figure S9. UV-Vis absorption spectrum of cobalt chloride 3 mM in MOPS 25 mM, NaCl 150 mM, DDM 0.01% pH 7.0.

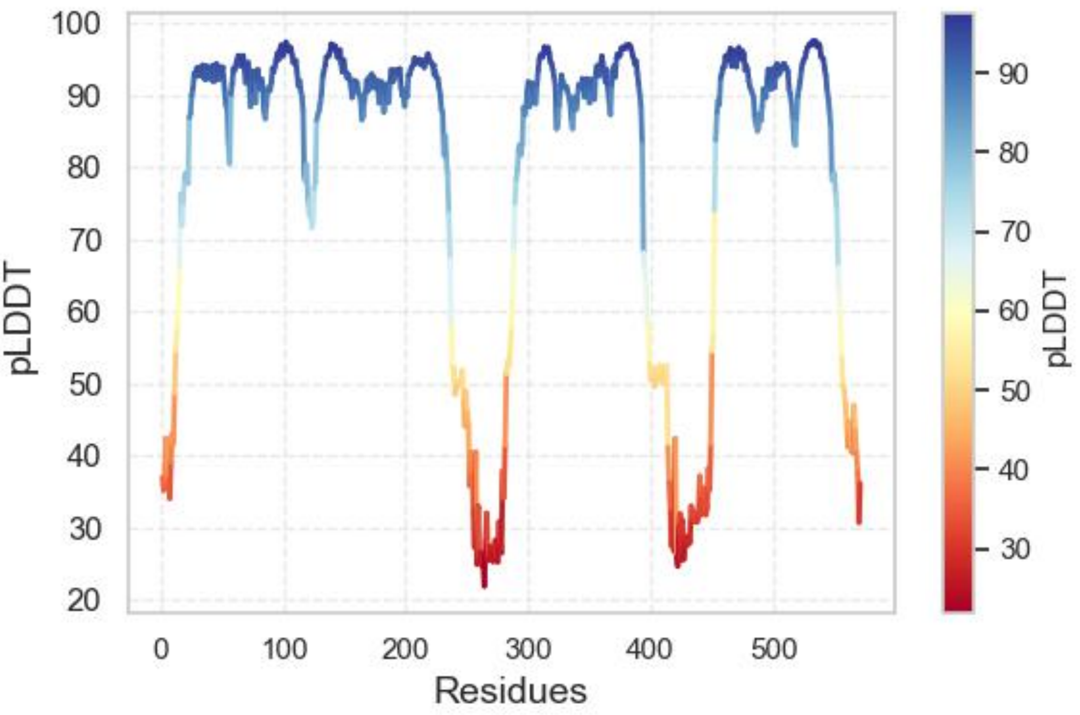

Figure S10. pLDDT plot for the inward-open model of Fpn.
